# Usage, acceptability, and preliminary effectiveness of an mHealth-based integrated modality for smoking cessation interventions in Western China

**DOI:** 10.18332/tid/156828

**Published:** 2023-01-21

**Authors:** Shuilian Chu, Zhaohui Tong, Yuntao Zhang, Xianwei Ye, Zhiyan Liu, Hong Chen, Jing Bai, Fengsen Li, Xiaoping Li, Huaizhen Wang, Rui Wang, Xuefeng Wang, Jiachen Li, Siqiao Liang, Ying Nong, Xin Wang, Ahong Wang, Di Zhang, Hang Jing, Lin Feng, Lirong Liang

**Affiliations:** 1 Department of Research on Tobacco Dependence Therapies, Beijing Institute of Respiratory Medicine, Beijing Chao-Yang Hospital, Capital Medical University, Beijing, China; 2 Department of Respiratory and Critical Care Medicine, Beijing Institute of Respiratory Medicine, Beijing Chao-Yang Hospital, Capital Medical University, Beijing, China; 3 Department of Respiratory Medicine, People’s Hospital of Lhasa Tibet, Lhasa, Tibet Autonomous Region, China; 4 Department of Pulmonary and Critical Care Medicine, Guizhou Provincial People’s Hospital, Guiyang, China; 5 Department of Respiratory and Critical Care Medicine, Xi'an Third Hospital, Xi'an, China; 6 Department of Pulmonary and Critical Care Medicine, The First Affiliated Hospital, Chongqing Medical University, Chongqing, China; 7 Department of Respiratory and Critical Care Medicine, The First Affiliated Hospital of Guangxi Medical University, Nanning, China; 8 Department of Respiratory and Critical Care Medicine, Affiliated Hospital of Traditional Chinese Medicine, Xinjiang Medical University, Urumqi, China; 9 Department of Respiratory and Critical Care Medicine, The First People’s Hospital of Zunyi, Zunyi, China; 10 Hospital Management Office, Kashgar Prefecture Second People’s Hospital, Kashgar, China; 11 Department of Respiratory and Critical Care Medicine, Xining Second People’s Hospital, Xining, China; 12 Department of Respiratory and Critical Care Medicine, Kashgar Prefecture Second People’s Hospital, Kashgar, China

**Keywords:** mobile health, smoking cessation, integrated modality, quitline, WeChat

## Abstract

**INTRODUCTION:**

Many smokers have not accessed professional smoking cessation assistance due to limited smoking cessation services. We developed a novel mHealth-based integrated modality for smoking cessation (WeChat + Quitline modality, WQ modality) and applied it to a large public welfare project (China Western-QUIT Program) in western China. This study evaluated the usage, acceptability, and preliminary effectiveness of the WQ modality in the population of western China.

**METHODS:**

A prospective cohort study was conducted between April and August 2021. Smokers or their relatives were recruited through online advertisements and medical staff referrals. After using the services of the WQ modality for one month, the self-reported awareness, use, and satisfaction with each service among the participants were collected by a telephone interview. We also evaluated the self-reported 7-day point prevalence of abstinence (PPA) and quit attempt rate among baseline current smokers. The usage data of each service were downloaded from quitline and WeChat platforms.

**RESULTS:**

Of the 17326 people from western China using the WQ modality, the largest number of users was WeChat official account (11173), followed by WeChat mini program (3734), WeChat group (669), and quitline (541 inbound calls, 605 outbound calls). At one month follow-up, over 70% of participants who completed the baseline survey (n=2221) were aware of WeChat-based services, and over 50% used them. However, the awareness rate (11.1%) and utilization rate (0.5%) of quitline were relatively low. The median satisfaction scores across all services were 9 out of 10 points (IQR: 8–9). Among the baseline current smokers (n=1257), self-reported 7-day PPA was 41.8% (526/1257), and another 225 smokers (17.9%) reported making a quit attempt.

**CONCLUSIONS:**

The WQ modality could be well used and accepted, and it has great potential to motivate and aid short-term smoking cessation in smokers from western China.

## INTRODUCTION

Quit smoking is the best measure to prevent and reduce smoking-related health hazards^1^. Due to the highly addictive nature of nicotine, it is necessary to provide smoking cessation interventions to help smokers in quitting^2^. However, many smokers have not accessed professional smoking cessation assistance^3^.

Barriers that prevent health professionals from providing cessation interventions include time constraints, a lack of knowledge and training, insufficient institutional support, a lack of adequate reimbursement for delivering tobacco treatment, and inadequate or confusing insurance cessation coverage^4^. An alternative cessation intervention approach (Ask-Advise- Refer) could remove these barriers^5^. It involves a provider in a clinical setting asking about tobacco use, advising patients to quit, and referring those who want to quit to other cessation resources, such as tobacco quitline, to receive more intensive cessation interventions^5^. However, due to limited resources in many countries and regions, it is difficult for physicians to refer smoking patients to professional cessation services for intensive treatment.

This limitation also exists in China, which has more than 300 million current smokers^6^, and approximately 1 million people die from smoking-related diseases each year^7^. As reported in Global Adult Tobacco Survey (GATS) China report 2018, more than 90% of Chinese smokers tried to quit smoking in the past 12 months without any professional help^6^. A nationwide survey showed that there were only 366 smoking cessation clinics among approximately 1 million hospitals in mainland China^8^. Furthermore, there are only three quitlines in mainland China. Two of them are nationwide quitlines run by hospitals (400-888-5531 in Beijing Chao-Yang Hospital, and 400-808-5531 in China-Japan Hospital), and the other one is a regional quitline integrated into a public health hotline (12320)^9^. They are all facing the challenges of low awareness and utilization due to a lack of financial support^9^. Therefore, it is imperative to develop accessible, affordable, and cost-effective delivery modalities to aid smokers in quitting.

Since offering non-face-to-face medical treatment without any limitations in terms of time or space, telemedicine can be a potential medical solution during the COVID-19 pandemic^10^. In the United States, from January to June 2020, 30.1% of medical visits were done via telemedicine^11^, and the weekly number of online medical visits increased 23-fold compared to the pre-pandemic period^12^. Telemedicine can also be used to help smokers quit. A variety of mobile health (mHealth) approaches for smoking cessation interventions have been developed and proven to be effective in assisting smokers to quit smoking, such as short message services, web-based interventions, and smartphone applications (apps)^13^. Although there are hundreds of Chinese smoking cessation apps available in the Apps Store, most of them have low levels of adherence to standard clinical practice guidelines^14^.

Given the dynamic, quickly evolving nature of the personal technology modalities used in mHealth, a possible strategy to improve their accessibility and efficacy is to integrate treatment from multiple sources so that a broader array of information and treatment options across multiple contexts could be integrated by professionals^5^. Therefore, we developed a novel intervention delivery modality for smoking cessation which integrated a professional quitline (400-888-5531) and three mHealth interventions based on an app (WeChat app, the most popular app in China^15^), including the QUIT mini program (similar to cessation apps), the QUIT online chat group (providing online cessation counseling and group intervention), and the QUIT official account (electronic self-help cessation materials), called the WeChat + Quitline modality (WQ modality). With this mode of delivery, we would be able to provide smokers with comprehensive mHealth-based smoking cessation services. As a major part of the World Health Organization (WHO) campaign for World No Tobacco Day 2021^16^, the China Western- QUIT program applied the WQ modality to deliver smoking cessation intervention in twelve provinces and cities of western China, where the smoking prevalence was relatively higher^6^ and cessation service resources are less available than in other parts of China^8^.

The purpose of this study was to evaluate the usage, acceptability, and preliminary effectiveness of the WQ modality in delivering smoking cessation interventions for the population from western China. In addition, we also aimed to inform future practice and research concerning integrated online cessation services delivered worldwide via mHealth technologies.

## METHODS

### Study design

Based on a prospective cohort study, the China Western-QUIT Program was implemented in western China from 20 April to 6 August 2021. The study protocol has been approved by the ethics committee of Beijing Chao-Yang Hospital, Capital Medical University (Ethics Approval No.: 2021-Ke-260, Date: April 12, 2021). Electronically informed consent was obtained from all participants before participating in the project.

### Participants and recruitment

Participants were recruited by advertising, media, free medical consultation, and medical staff referrals. Eligibility criteria were: 1) smokers or their relatives, living in western China; 2) aged ≥18 years; 3) completed the baseline survey; and 4) willing to receive a follow-up at one month. Smokers were those who had smoked tobacco products in their lifetime, and current smokers were those who were currently smoking at baseline^17^. In addition, 244 medical staff from nine hospitals in western China (Kashgar, Urumqi, Lhasa, Xi’an, Xining, Chongqing, Guizhou, Zunyi, and Nanning) engaged in recruitment.

### Interventions

**Figure 1:**
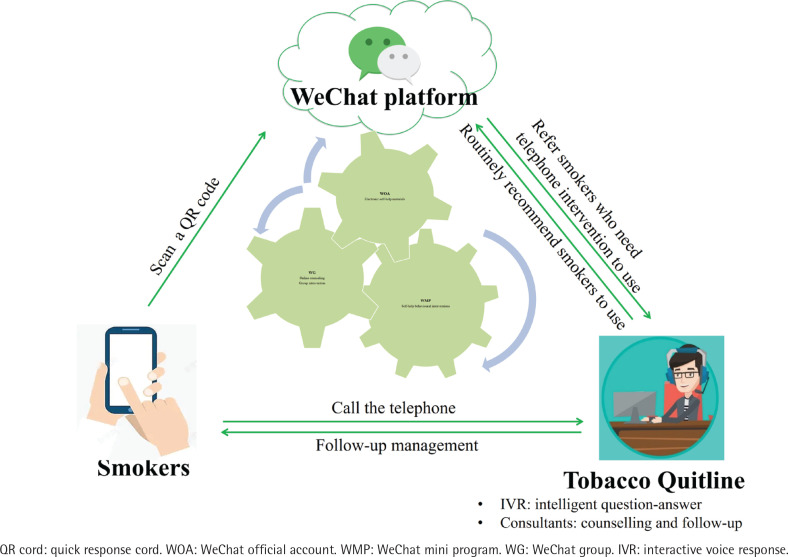
The components of WeChat & Quitline modality applied in western China in 2020 and the connection between the components

**Figure 2:**
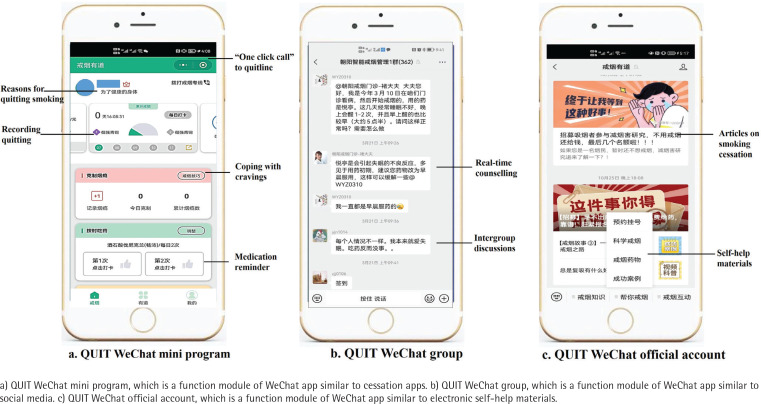
Screenshots of WeChat-based interventions of WeChat & Quitline modality applied in western China in 2020

As shown in Figure 1, the WQ modality integrated a professional quitline (400-888-5531) and three WeChat-based cessation services, including the QUIT WeChat mini program (WMP), the QUIT WeChat group (WG), and the QUIT WeChat official account (WOA). The screenshots of the WeChat-based cessation services are shown in Figure 2.

Quitline services include 24-hour Interactive Voice Response (IVR)-based counselling, reactive counselling from 8:30 a.m. to 5:30 p.m., proactive behavior interventions (3–5 sessions) and short message service^18^. The QUIT WMP is an application function module embedded in WeChat app, which could assist smokers in building skills for quitting and motivating them to do so. It was developed by using the behavior change wheel framework^19^ and the clinical practice guideline for smoking cessation^20^. The QUIT WG could provide online group interventions, including real-time counseling from smoking cessation specialists and intergroup discussions, etc. The QUIT WOA could provide internet-based self-help materials for smoking cessation, including articles, videos, and other materials.

All of these services are integrated and connected. The QUIT WOA is the main entry point, creating an introduction, and links to other services automatically. The medical assistants in the QUIT WG will recommend, guide and supervise smokers to use the QUIT WMP, and refer smokers to call quitline for assistance. In the QUIT WMP, links to other three services were provided, including a feature called ‘One Click Call Quitline’. Quitline consultants will also routinely recommend smokers to use WeChat-based services after counselling.

### Data collection

Baseline data were collected via an online questionnaire, including demographic information, smoking characteristics, and comorbidities. Nicotine dependence was measured by the Fagerström test for nicotine dependence (FTND)^21^.

Participants were followed up by telephone at one month after baseline. Those recruited by medical staff were followed up by medical staff, and others were done by quitline consultants. An online questionnaire was used to collect information about the awareness of, use of, and satisfaction with the WQ modality among all participants and the cessation behaviors among baseline smokers. A scale of 0 to 10 (0: not at all; 10: very high), satisfaction with each service was evaluated. For baseline current smokers, smoking status was measured by the question: ‘Have you smoked cigarettes, even a puff, during the past seven days?’. Quitting attempt was measured by the question: ‘Have you tried to quit smoking for at least 24 hours in the past month?’.

An online survey was designed to determine the adoption of the WQ modality among medical staff. Service usage data during the project period were downloaded from WeChat and quitline platforms.

### Outcomes

The usage outcomes included the cumulative number of users, and the corresponding usage data during the project period downloaded from WeChat and quitline platforms.

The acceptability outcomes included self-reported awareness, use, and satisfaction with each service among the participants. In addition, we also evaluated the acceptability of referring patients to use the WQ modality among participating medical staff, including self-reported time spent and work burden associated with referral as well as their willingness to do so.

The primary effectiveness outcome was self-reported 7-day point prevalence of abstinence (PPA)^22^, defined as the proportion of smokers who reported not smoking in the past 7 days at follow-up at one month. The secondary effectiveness outcome was the quit attempt rate, defined as the proportion of smokers who were continuously smoking but have made at least one quit attempt (more than 24 hours^23^) in the past month. Due to the COVID-19 pandemic, participants were unwilling to visit the hospital, and their smoking status was not biochemically validated. Nevertheless, 20% of baseline current smokers who completed the follow-up were randomly selected to be contacted again within one week to verify their smoking status.

### Statistical analysis

All statistical analyses were performed using Stata, version 15.0 (Stata Corp). In descriptive analyses, the frequency and percentages of categorical variables, and the means and standard deviations of continuous variables are presented. At baseline, smokers were divided into three groups according to their willingness and the plan to quit: without willingness, with willingness but no plan, and with plan to quit. We compared the 7-day PPA and quit attempt rate at one month between the three groups by χ^2^ test, and used logistic models to calculate the p-trend. Logistic regression was also used to analyze the relationship between 7-day quitting and using different cessation services, with the odds ratios (OR) and 95% confidence intervals (CIs). The percentages of increase in the probability of 7-day quitting in the exposure group compared with the control group were calculated by multiplying (OR-1) by 100%. Participants who were lost to follow-up were classified as continuous smokers. All statistical tests were two-tailed with a significance level of 0.05.

## RESULTS

### Project reach

**Figure 3:**
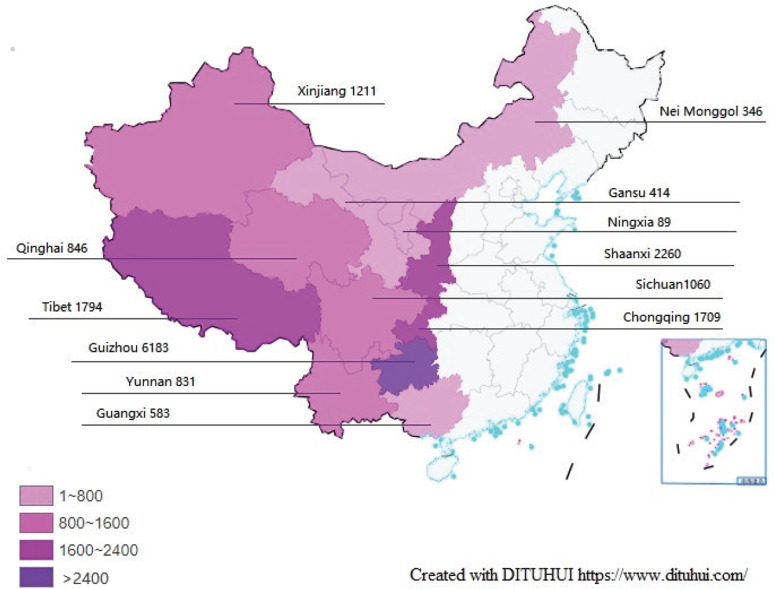
Participants distribution by province, autonomous region, and municipality of the China-Western QUIT Program in 2020 (N=17326)

**Figure 4:**
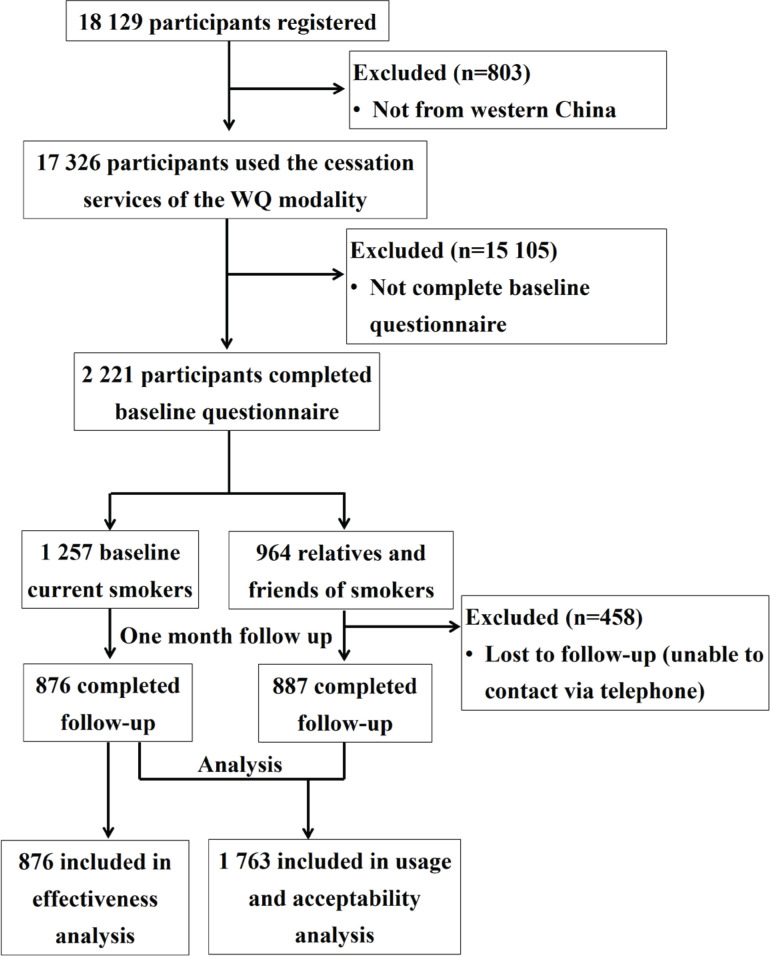
Scheduling of recruitment and follow-up of the China-Western QUIT Program in 2020 (N=17326)

From 20 April to 6 August 2021, a total of 18129 people used the services of the WQ modality, and 17326 of them were from western China. The regional distribution of participants is shown in Figure 3. Only 2221 participants (including 1257 current smokers) filled in the baseline questionnaire and were included in the final analysis. Follow-up at one month was completed by 1763 participants (79.4%), including 876 baseline current smokers and 887 smokers’ relatives (Figure 4). The participants that lost follow-up were mostly men, older, less educated, engaged in manual work, unemployed, with lower income, former smokers, and had fewer comorbidities (Supplementary file Table 1).

### Participants’ characteristics

Among participants who completed the baseline questionnaire (n=2221), 73.0% were men, the mean age was 36.3 ± 11.8 years, 70.0% had a college degree or higher, and 27.1% with comorbidities. The baseline characteristics of current smokers (n=1257) were similar to the overall participants. However, current smokers were more likely to be men (86.1%) and had a relatively higher prevalence of comorbidities (31.4%). On average, the baseline current smokers had smoked for 15 years, smoked 20 cigarettes per day, and 68.5% of them were moderate or severe nicotine dependent (FTND score ≥4). Nearly two-thirds of current smokers had tried to quit smoking, and 75.9% were willing to quit smoking, of which 44.3% planned to quit smoking within one month (Table 1).

**Table 1:** Demographic and smoking characteristics, and comorbidities of participants, western China, 2020 (N=2221)

	*Overall (n=2221) n (%)*	*Current smokers (n=1257) n (%)*
**Demographic characteristics**		
**Sex**		
Male	1621 (73.0)	1082 (86.1)
Female	600 (27.0)	175 (13.9)
**Age** (years), mean ± SD	36.3 ± 11.8	37.5 ± 11.2
18–24	189 (8.5)	75 (6.0)
25–44	1486 (66.9)	852 (67.8)
45–64	493 (22.2)	296 (23.5)
≥65	53 (2.4)	34 (2.7)
**Education level**		
Middle school and lower	337 (15.2)	213 (16.9)
High school	330 (14.9)	190 (15.1)
College and higher	1554 (70.0)	854 (67.9)
**Occupation**		
Technical staff	575 (25.9)	294 (23.4)
Business/service staff	457 (20.6)	301 (23.9)
Administrative staff	436 (19.6)	247 (19.6)
Workers/farmers	359 (16.2)	236 (18.8)
Students/freelancers/retirees	252 (11.3)	94 (7.5)
Other	142 (6.4)	85 (6.8)
**Household income per year** (RMB)		
≤100000	1558 (70.2)	851 (67.7)
>100000	663 (29.8)	406 (32.3)
**Smoking characteristics**		
**Smoking status**		
Never smokers	728 (32.8)	
Former smokers^a^	236 (10.6)	
Current smokers^b^	1257 (56.6)	
Occasional smokers	289 (13.0)	289 (23.0)
Daily smokers	968 (43.6)	968 (77.0)
Cigarettes per day, median (IQR)		20.0 (10.0–20.0)
Smoking duration (years), median (IQR)		15.0 (10.0–20.0)
**FTND^c^ score**		
0–3		396 (31.5)
4–6		528 (42.0)
7–10		333 (26.5)
**Prior quit attempt**		
Yes		783 (62.3)
No		474 (37.7)
**Used quitting aid** (n=671)		
‘Cold turkey’		485 (72.3)
E-cigarette		119 (17.7)
Counseling		104 (15.5)
Pharmacotherapy^c^		85 (12.7)
Quitline		20 (3.0)
Traditional Chinese medicine		18 (2.7)
Other		55 (8.2)
**Willing to quit** (n=1257)		
Yes		954 (75.9)
No		303 (24.1)
**Plan to quit within 30 days** (n=954)		
Yes		423 (44.3)
No		531 (55.7)
**Comorbidities**		
At least one comorbidity	601 (27.1)	395 (31.4)
Hypertension	203 (9.1)	140 (11.1)
COPD^d^	161 (7.2)	115 (9.2)
Hyperlipemia	150 (6.7)	115 (9.2)
Asthma	80 (3.6)	48 (3.8)
Diabetes	79 (3.6)	53 (4.2)
Coronary heart disease	58 (2.6)	36 (2.9)
Cancer	35 (1.6)	18 (1.4)
Stroke	34 (1.5)	16 (1.3)
Other	105 (4.7)	63 (5.0)

a Former smokers: those who had smoked and quit smoking more than 30 days^4^.

b Current smokers: those who smoked at the time of the survey^4^.

c FTND: Fagerström test of nicotine dependence^17^.

d Pharmacotherapy including nicotine replacement therapy, varenicline, and bupropion.

e Chronic obstructive pulmonary disease. RMB: 1000 Chinese Renminbi about US$146.

### Use of smoking cessation services

According to the usage data obtained from WeChat and quitline platforms during the project period, the number of users on WOA was the highest (11173), followed by WMP (3734), WG (669), and quitline (inbound and outbound calls were 541 and 605, respectively). There were only a few participants that used multiple services (553/17326; 3.2%), of which the majority used a combination of WeChat-based services (393/553; 71.1%). In May 2021, when medical staff were involved in recruitment, the number of daily new users and cumulative users soared (Supplementary file Figures 1 and 2).

According to the usage data self-reported by participants at follow-up at one month, a high proportion of participants reported knowing about and using WOA (knew: 89.8%, used: 72.9%), WMP (knew: 81.1%, used: 58.9%), and WG (knew: 71.7%, used: 51.6%), while fewer participants reported being aware of quitline (400-888-5531) (11.1%), and even fewer participants reported having called (0.5%) or received a call (5.5%) from this quitline. Among 876 baseline current smokers, 716 (81.7%)

reported that they had used the cessation service based on the WQ modality (WOA: 679, WMP: 529, WG: 452, Quitline: 56), of which 441 (50.3%) had used the WQ modality combined with other cessation interventions (counselling in smoking cessation clinics: 287; cessation medications: 108; electronic cigarettes: 153).

### Acceptability of the WQ modality

The median satisfaction score of each service among participants was 9 out of 10 points (IQR: 8–9). One hundred and fifty medical staff completed the survey on the adoption of the WQ modality. In referring smokers to use these services, most medical staff reported that they would spend no more than 3 minutes (78.0%), it would not increase their workload (89.3%), and that they were willing to do so in the future (98.7%).

### Preliminary effectiveness of the WQ modality

Among the 1257 baseline current smokers, 7-day PPA at one month was 41.8% (526/1257), and the quit attempt rate was 17.9% (225/1257) (Table 2). The smoking status of 175 randomly selected smokers was verified by an additional telephone, and the consistency rate was 76.0% (133/175).

**Table 2:** Participants’ self-reported 7-day quitting and quitting attempt at follow-up at one month, western China, 2020 (N=1257)

	*n*	*Self-reported smoking status at follow-up at 1 month, n (%)*			*p*
		*Continued smoking*	*Quitting attempt*	*7-day quitting*	
**Overall**	1257	506 (40.3)	225 (17.9)	526 (41.8)	
**Willingness and plan to quit at baseline**					0.070
No willingness to quit	303	128 (42.2)	45 (14.9)	130 (42.9)	
Willingness but no plan to quit	531	221 (41.6)	86 (16.2)	224 (42.2)	
Plan to quit	423	157 (37.1)	94 (22.2)	172 (40.7)	
p		0.141	**0.007**	0.533	

To evaluate the impact of baseline willingness and plan to quit smoking on cessation behaviors, we conducted a stratified analysis (Table 2). There was a lack of clear evidence to find the difference in 7-day PPA between smokers with different willingness to quit (42.9% vs 42.2% vs 40.7%, p=0.533). But the quit attempt rate in smokers who planned to quit (22.2%) was higher than in those with no willingness (14.9%) or those with willingness but no plan to quit (16.2%) (p=0.007).

To explore the effect of using different services based on the WQ modality on baseline smokers’ self-reported 7-day quitting at follow-up at one-month, we conducted a multivariable logistic regression analysis (Table 3). After adjusting for sex, age, education level, household income per year, and the use of other interventions, compared with smokers who did not use any services, the likelihood of 7-day quitting at one month among those who used the WMP or WG increased by 118% (AOR=2.18; 95% CI: 1.57–3.04) and 193% (AOR=2.93; 95% CI: 2.11–4.07), respectively. However, no significant association was found for using the WOA (AOR=1.26; 95% CI: 0.86–1.84) and the quitline (AOR=0.69; 95% CI: 0.35–1.34). In addition, a dose-response relationship was present between the number of services used and the proportion of smokers reporting 7-day quitting at one month (p<0.001). The most effective combination of two or more services was the WOA+WMP+WG (AOR=1.95; 95% CI:1.21–3.15).

**Table 3:** Association between using different cessation services and participants’ self-reported 7-day quitting at follow-up at one month, western China, 2020 (N=876)

	*n*	*7-day quitting*	*AOR (95% CI)*	*p*
**Type of service**				
QUIT WOA^b^ (vs none)	679	347 (51.1)	1.26 (0.86–1.84)	0.230
QUIT WMP^c^ (vs none)	529	309 (58.4)	2.18 (1.57–3.04)	**<0.001**
QUIT WG^d^ (vs none)	452	290 (64.2)	2.93 (2.11–4.07)	**<0.001**
Quitline (vs none)	56	19 (33.9)	0.69 (0.35–1.34)	0.273
**Number of services**				
None (Ref.)	160	56 (35.0)	1	
Used any 1	159	49 (30.8)	0.53 (0.31–0.91)	**0.021**
Used any 2	135	35 (25.9)	0.57 (0.33–1.00)	0.051
Used any 3	401	270 (67.3)	2.10 (1.34–3.28)	**0.001**
Used all 4	21	9 (42.9)	2.32 (0.79–6.77)	0.125
p-trend				**<0.001**
**Different combinations of two or more services**				
**WeChat**				
None (Ref.)	160	56 (35.0)	1	
WOA+WMP	88	25 (25.5)	0.53 (0.28–1.02)	0.059
WOA+WG	20	9 (29.0)	0.56 (0.21–1.51)	0.255
WOA+WMP+WG	393	271 (68.3)	1.95 (1.21–3.15)	**0.006**
p-trend				**<0.001**
**WeChat+quitline**				
None (Ref.)	160	56 (35.0)	1	
Only WeChat-based services	660	344 (52.1)	1.16 (0.77–1.75)	0.720
Only quitline	4	1 (25.0)	1.28 (0.12–13.35)	0.200
WeChat-based services plus quitline^e^	52	18 (34.6)	0.75 (0.35–1.62)	0.463
p-trend				0.628

AOR: adjusted odds ratio; adjusted for age, sex, education level, occupation, household income per year, and using other interventions including counselling in smoking cessation clinics, using cessation medications or electronic cigarettes.

b WOA: WeChat official account.

c WMP: WeChat mini-program.

d WG: WeChat group.

e WeChat-based services plus quitline include WOA+quitline (n=15), WOA+WMP+quitline (n=5), WOA+WG+quitline (n=3), WOA+WMP+WG+quitline (n=21), and WMP+quitline (n=8).

## DISCUSSION

This study evaluated the usage, acceptability, and preliminary effectiveness of the WQ modality in western China, which is a novel mHealth-based integrated modality for smoking cessation interventions. The results showed that most participants were willing to use these services, and were highly satisfied with them. After using the services of the WQ modality, nearly half of baseline current smokers reported that they had successfully quit smoking, and the more services used the more likely to quit.

The results demonstrated that the WQ modality could be readily used and accepted. Two possible reasons could explain the extensive usage and good acceptability of the WQ modality. Firstly, the WQ modality integrated four mHealth-based approaches, which could remove the barriers to accessing cessation services (e.g. barriers related to scheduling and transportation), thereby leading to widespread use^5^. Our results also suggested that the services of the WQ modality were very attractive to smokers and their relatives in western China. Before applying the WQ modality, the number of users in the WQ modality was more than five times the annual call volume of the quitline 400-888-5531^18,24^. High engagement aligns made more and more people interested in using mHealth to access medical care^10-12^. Secondly, the WeChat app was chosen to integrate with quitline due to its high popularity among Chinese^25,26^. Moreover, it integrates multiple functions into one platform, such as WOA, WMP, and WG^15^. This way, comprehensive smoking cessation services can be provided through one app.

Moreover, the WQ modality has the potential of assisting smokers in quitting. The self-reported 7-day PPA at one month follow-up among baseline smokers in our study was slightly lower than that of a smoking cessation WeChat mini program in a pilot randomized controlled trial (RCT) (63%)^27^, and higher than that of a quitline service in China (27.4%)^28^. The positive effect of the WQ modality is related to its integration of multiple evidence-based effective smoking cessation interventions. Quitline is an essential part of the WQ modality, which could provide personal counselling and proactive behavior interventions, and a large amount of evidence supports the effectiveness of quitline^29^. Similar to cessation apps, the QUIT WMP could provide behavioral interventions to help smokers build skills to quit, and many studies have demonstrated the effectiveness of cessation apps in assisting smokers to quit^30-32^. Moreover, the QUIT WMP was developed using the behavior change wheel framework^19^ and the clinical practice guideline for smoking cessation^20^, which enhanced the effectiveness of the intervention provided by the QUIT WMP. Essentially, WMP is the evolution of mobile apps, which could be accessed by scanning a quick response (QR) code or searching directly within the WeChat app without downloading or installing^15^, which also eliminates some limitations of the smartphone apps. A pilot RCT found that delivering cessation interventions through WMP may be effective and feasible for assisting smokers in quitting^27^. The QUIT WG provided online group intervention for at least 100 days. Professionally led, group-based treatment yielded high quitting rates^33^. The QUIT WG could remove the barrier of participating in face-to-face group interventions. More importantly, it facilitates the formation of mutually reciprocated, strong, and long-lasting social bonds that support smoking cessation in a similar manner to that of Twitter and Facebook^34-36^. The QUIT WOA provides non-tailored electronic smoking cessation self-help material, updated regularly and providing a lot of smoking cessation information. Evidence indicates that non-tailored self-help materials for smoking cessation must combine with in-person or technology-based interventions^5^. Consistent with this, our results showed that when combined with quitline and WeChat-based services, the QUIT WOA could encourage smokers to quit. All of these services are integrated and connected, which enables smokers to obtain more comprehensive interventions and enhance the effectiveness of smoking cessation interventions. Additionally, the high abstinence rate in this study might be related to more baseline smokers using the WQ modality together with other smoking cessation interventions, such as counselling among smoking cessation clinics and cessation medications, which was due to media publicity and medical staff referrals during the project period.

We found that compared with WeChat-based services, quitline has the lowest utilization rate, which indicated that quitline was not popular with Chinese people. This was consistent with other studies. In the United States, even among smokers who tried to quit and were aware of quitlines, the reach rate was only around 8%^37^. In China, quitlines are also facing similar challenges^9^. For example, from December 2009 to May 2012, there were only 8260 callers of quitline 400-888-5531^24^, and inbound calls decreased from 215 per month in 2016 to 170 per month in 2018^18^. A large part of the lower reach and use of quitline is due to limited funding, which in turn leads to fewer publicity activities and unsatisfactory service^37^. There is a lack of financial support for quitlines in China. There are two nationwide quitlines (400-888-5531 and 400-808-5531) supported by limited research funds. Other regional quitlines operated part-time by the local public health hotline (12320) were also underfunded. Another possible reason might be that more and more people prefer to communicate using the chat tools on their smartphones instead of the telephone^38^. Therefore, the service mode of quitline should evolve to meet user preferences. In 2014, Minnesota implemented a modality that offered more choices of cessation services, including quitline counselling, an NRT starter kit, text messaging, etc., which had the potential to increase the use of the quitline^39^. Another possible strategy is integrating quitline with other cessation services^5^. The rapid development of mHealth technology makes it possible to integrate quitline with mHealth platforms. The clinical practice guideline also recommended integrating cessation services across multiple platforms and within healthcare systems^5^. This will not only help to transfer information between different platforms, but also encourage mutual referral between services, thus increasing the reach and usability, and improving the effectiveness of interventions^5^. As far as we know, the WQ modality is the first reported integrated modality for delivering comprehensive smoking cessation services.

However, we found several problems with the WQ modality during the project period. Firstly, the use of the QUIT WMP decreased markedly with the end of the project. The evidence indicates a dose-response relationship between the intensity of behavioral therapy and the success of quitting^5^. Hence, the problem remains how to improve the long-term compliance of the QUIT WMP. Secondly, fewer people were active in the QUIT WG during the project period, partly due to the research team lacking experience in managing the group effectively. Further research is still needed to increase the understanding of the potential, benefits and limitations of WG for smoking cessation. Thirdly, the proportion of combined use services is relatively low (553/17326; 3.2%), which indicates that more effective strategies need to be developed to facilitate the combined use of multiple services. These findings could be further used as guidance to improve this modality.

In addition, it is worth noting that nearly two-thirds of smokers who were unwilling to quit at baseline reported quitting in the past seven days (130/303; 42.9%) or tried to quit smoking (45/303; 14.9%) at one month follow-up. The intriguing finding may be attributed to multiple factors. Firstly, it is suggestive, but not sufficient to infer that WQ modality can improve the success rate of smoking cessation. Secondly, the medical staff played a vital role in bringing about the change. Both the WHO and the clinical practice guidelines emphasize the importance of quit advice from medical staff in encouraging smokers to quit^2,5^, and patients respect and trust medical staff^40^. Therefore, if the medical staff advise smokers to quit smoking, smokers are more likely to quit smoking and use cessation services. This provides a potential way to generalize the WQ modality, combining it with a brief smoking cessation intervention based on the health information system^41^, and it may also become a possible smoking cessation service resource for medical staff referrals. Finally, it may be partly explained by the extensive media coverage of the WQ modality during the project period, especially World No Tobacco Day’s extensive publicity campaigns. There is evidence that mass media campaigns can effectively motivate smokers to quit at the population level^42^. Therefore, our findings suggest that a combination of medical staff and publicity activities will be beneficial in generalizing the WQ modality in a wider area of China. Although we did not use a nationally representative sample, the participants were from 12 different provinces across China, which increased the generalizability of the study results. However, it still needs financial support to maintain its implementation, such as regularly updating the WeChat official account and hiring consultants for the WeChat groups. Therefore, it is necessary to research the cost-effectiveness of this modality in the future.

### Limitations

There are several limitations. Firstly, as the China Western-QUIT Program was an important part of the WHO campaign for World No Tobacco Day in China, we evaluated the WQ modality through a prospective cohort study rather than an RCT, so our conclusions remain preliminary. Secondly, time constraints forced us to assess the 7-day PPA at one month as the primary effectiveness outcome. It is unclear whether the WQ modality will affect long-term smoking cessation. Thirdly, the information on cessation status was based on self-report, which was not biochemically validated. Inevitably, this may have resulted in a reporting bias in smokers^43^, and thus abstinence rates were overestimated. Therefore, it is necessary to confirm the effectiveness of the WQ modality in well-conducted RCT. Additionally, since more than 70% of participants were men, young to middle-aged, and highly educated, it is important to extrapolate the results with caution to subgroups such as women, the elderly, and those with low literacy. Despite these limitations, the WQ modality offers a promising direction for integrating cessation services across multiple platforms and within healthcare systems, thereby expanding its reach and use. More importantly, this modality provides an essential reference for addressing the challenge of smoking cessation services during the ongoing pandemic of COVID-19.

## CONCLUSIONS

The WQ modality could be well used and accepted in the population of western China. It has the potential to achieve broad coverage and may motivate and aid short-term smoking cessation in western China. Further research is needed to provide a more conclusive understanding of this modality.

## Data Availability

The data supporting this research are available from the authors on reasonable request.
