# Supplementary material for: Usage, acceptability, and preliminary effectiveness of an mHealth-based integrated modality for smoking cessation interventions in Western China
Source: Tob Induc Dis. 2023 Jan 21;21:07. doi: 10.18332/tid/156828 (PMC9865639; doi:10.18332/tid/156828)
Supplement: Supplementary file 1 [file TID-21-07-s1.pdf]

Supplementary Table 1. Comparison of baseline characteristics between participants lost and completed follow-up

	Lost follow-up (N=458)	Completed follow-up (N=1 763)	*P*
**Demographic characteristics**			
Sex, n (%)			<0.001
Male	372 (81.2)	1252 (71.0)	
Female	86 (18.8)	511 (29.0)	
Age (years), mean ± SD	36.3±11.8	37.5±11.2	0.008
18-24	44 (9.6)	145 (8.2)	
25-44	279 (60.9)	1204 (68.3)	
45-64	119 (26.0)	376 (21.3)	
≥65	16 (3.6)	38 (2.2)	
Education, n (%)			<0.001
Middle school and below	87 (19.0)	243 (13.8)	
High school	69 (15.1)	257 (14.6)	
College and above	302 (65.9)	1263 (71.6)	
Occupation, n (%)			<0.001
Technical staff	109 (23.8)	464 (26.3)	
Business/ service staff	87 (19.0)	370 (21.0)	
Administrative staff	61 (13.3)	372 (21.1)	
Workers/ farmers	91 (19.9)	277 (15.7)	
Students/ freelancers /retirees	72 (15.7)	176 (10.0)	
Other	38 (8.3)	104 (5.9)	
Household income per year (¥), n (%)			<0.001
≤100 000	331 (72.3)	1229 (69.7)	
>100 000	127 (27.7)	534 (30.3)	
Smoking status, n (%)			<0.001
Never smokers	145 (31.7)	569 (32.3)	
Former smokers	64 (14.0)	160 (9.1)	

	Lost follow-up (N=458)	Completed follow-up (N=1 763)	*P*
Occasional smokers	41 (8.9)	272 (15.4)	
Daily smokers	208 (45.4)	762 (43.2)	
Comorbidities			
With at least one comorbidity, n (%)	120 (26.2)	481 (27.3)	<0.001
Hypertension, n (%)	39 (8.5)	164 (9.3)	0.603
COPD [a], n (%)	35 (7.6)	126 (7.2)	0.716
Hyperlipaemia, n (%)	34 (7.4)	116 (6.6)	0.521
Asthma, n (%)	18 (3.9)	62 (3.5)	0.672
Diabetes, n (%)	16 (3.5)	63 (3.6)	0.934
Coronary heart disease, n (%)	9 (2.0)	49 (2.8)	0.330
Cancer, n (%)	6 (1.3)	29 (1.6)	0.608
Stroke, n (%)	9 (2.0)	25 (1.4)	0.396
Other, n (%)	26 (5.7)	79 (4.5)	0.283

[a] Chronic obstructive pulmonary disease

Supplementary Figure 1 A, B and C

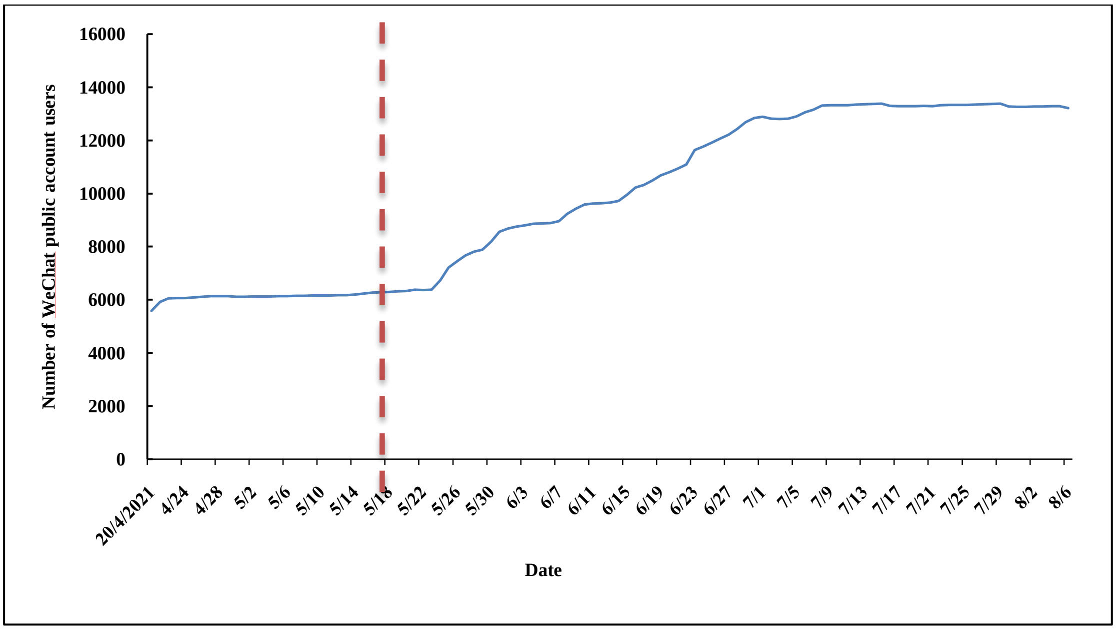

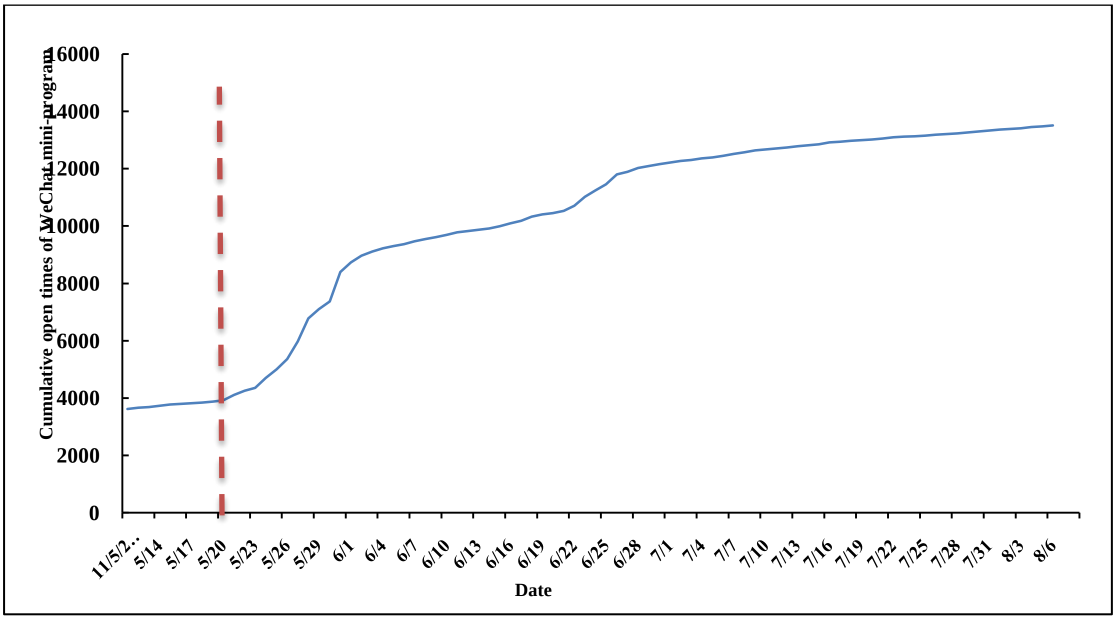

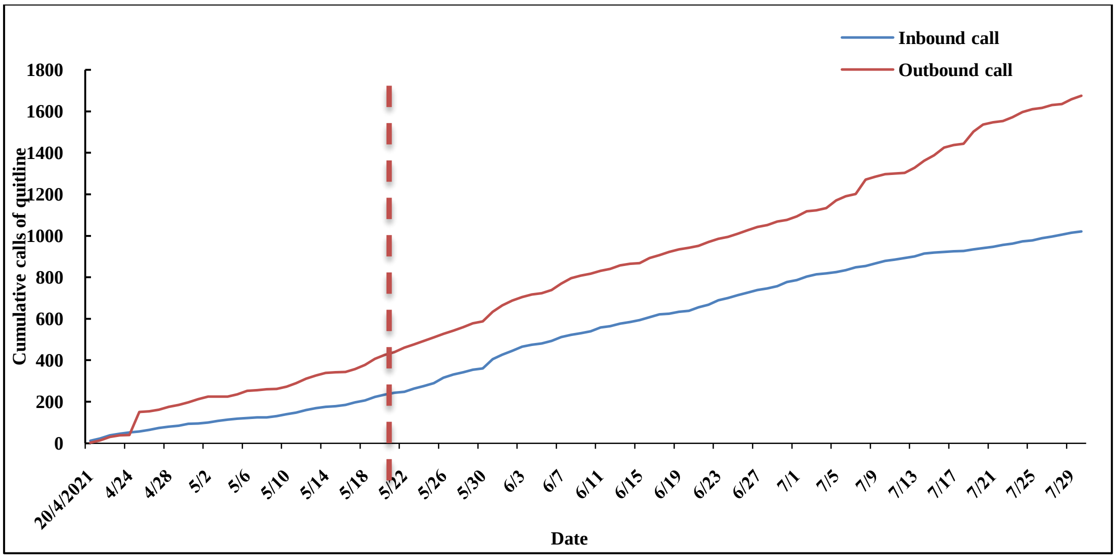

Supplementary Figure 2 A and B

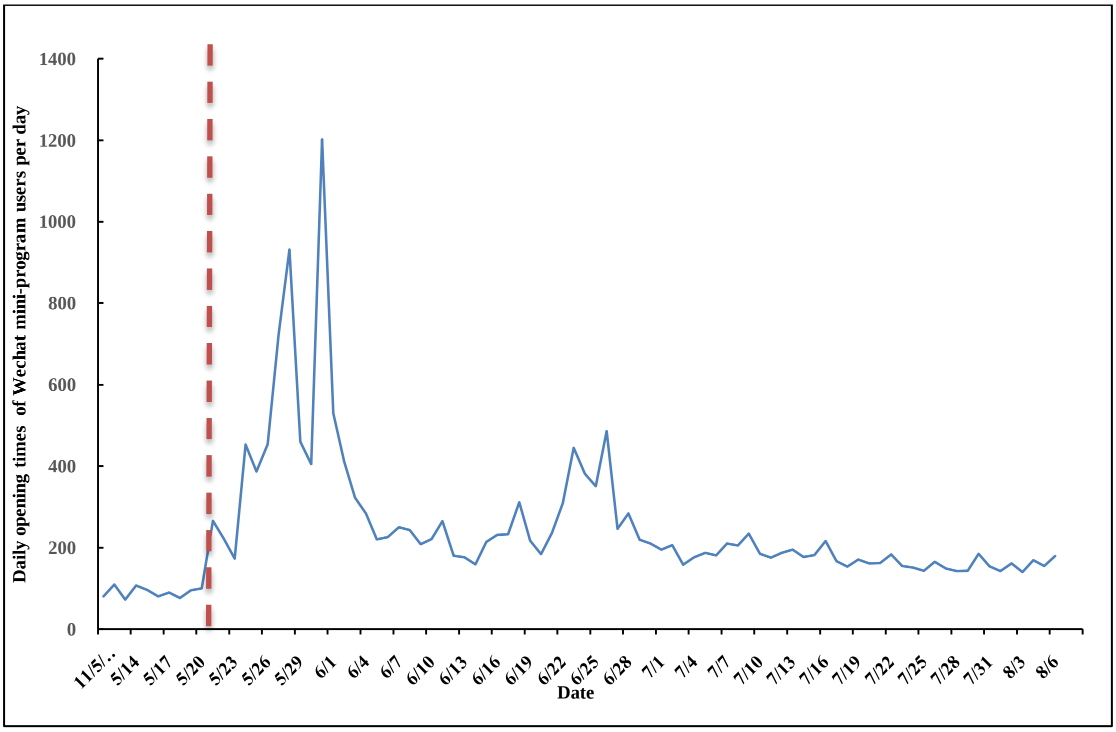

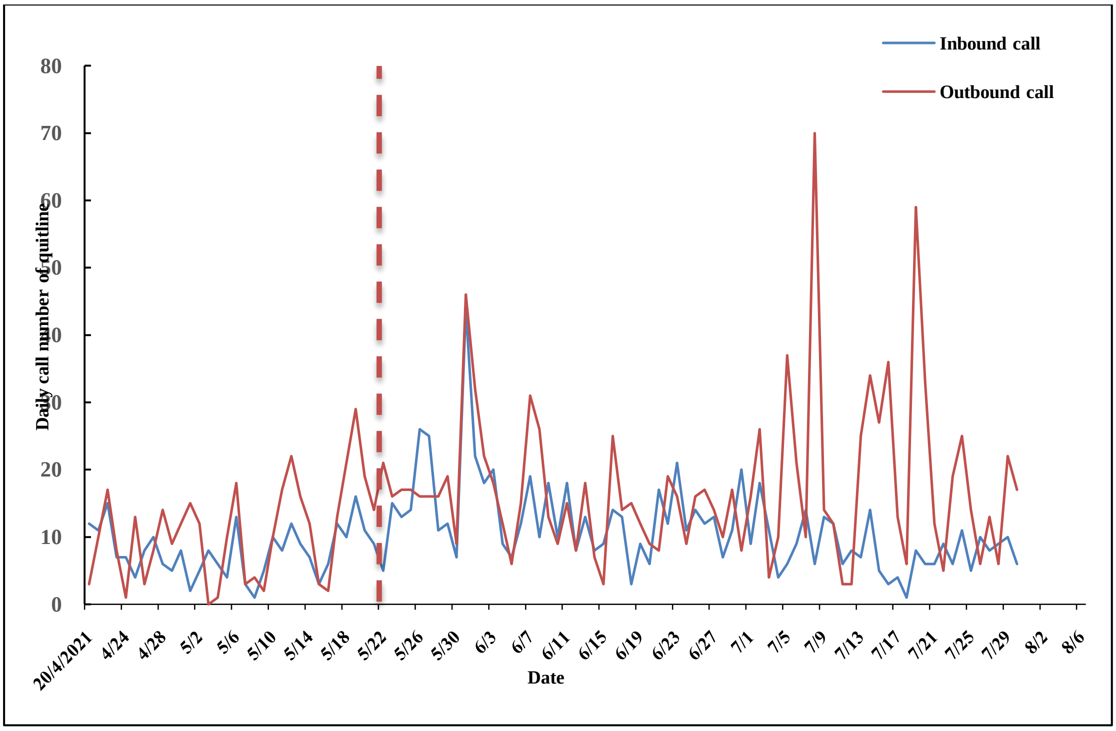

© 2023 Chu S. et al.
